# Brazilian obstetrician-gynecologists and abortion: a survey of knowledge, opinions and practices

**DOI:** 10.1186/1742-4755-2-10

**Published:** 2005-11-15

**Authors:** Lisa A Goldman, Sandra G García, Juan Díaz, Eileen A Yam

**Affiliations:** 1 University of California, Berkeley, Department of Epidemiology, 140 Warren Hall #7360, Berkeley, CA 94720, USA; 2 Population Council, Regional Office for Latin America and the Caribbean, Panzacola #62-102, Col. Villa Coyoacán, Mexico City 04000, Mexico; 3 Population Council, Brazil Office, Caixa Postal 6509, Cidade Universitária, Campinas, São Paulo, Brazil, Cep. 13084-970, Brazil

## Abstract

**Background:**

Abortion laws are extremely restrictive in Brazil. The knowledge, opinions of abortion laws, and abortion practices of obstetrician-gynecologists can have a significant impact on women's access to safe abortion.

**Methods:**

We conducted a mail-in survey with a 10% random sample of obstetrician-gynecologists affiliated with the Brazilian Federation of Obstetricians and Gynecologists. We documented participants' experiences performing abortion under a range of legal and illegal circumstances, and asked about which abortion techniques they had experience with. We used chi-square tests and crude logistic regression models to determine which sociodemographic, knowledge-related, or practice-related variables were associated with physician opinion.

**Results:**

Of the 1,500 questionnaires that we mailed out, we received responses from 572 (38%). Less than half (48%) of the respondents reported accurate knowledge about abortion law and 77% thought that the law should be more liberal. One-third of respondents reported having previous experience performing an abortion, and very few of these physicians reported having experience with manual vacuum aspiration (MVA) or with misoprostol with either mifepristone or methotrexate. Physicians that favored liberalization of the law were more likely to have correct knowledge about abortion law, and to be in favor of public funding for abortion services.

**Conclusion:**

Brazilian obstetrician-gynecologists need more information on abortion laws and on safe, effective abortion procedures.

## Background

In Brazil, as in most of the Latin America and Caribbean (LAC) region, abortion is highly legally restricted. The Brazilian Penal Code dating back to 1940 states that abortion is illegal except when performed to save a woman's life or in the case of rape. Although not explicitly permitted by law, abortions in the case of fetal malformation incompatible with neonatal life can be approved on a case by case basis by judicial discretion, a process that requires a lawyer's petition and statements by three physicians and a mental health professional. The law does not establish a legal gestational age limit; however, Ministry of Health guidelines recommend that abortions be conducted before 12 weeks gestation. Despite legal limitations, each year an estimated 1.4 million clandestine abortions are performed in Brazil resulting in some 300,000 hospitalizations for complications [1,2]. Unsafe abortion represents the third leading cause of maternal mortality, and deaths from unsafe abortion in Brazil comprise 12% of the maternal mortality ratio [1,3]. Brazil's abortion rate is high at 40.8 per 1,000 women, with approximately 31% of all pregnancies ending with an induced abortion [4,5].

In July 2004, a lone Brazilian federal judge issued a preliminary ruling that waived the requirement for court authorization for abortions in cases of fetuses with anencephaly (a fatal congenital defect that prevents formation of the brain), unleashing a fervent national debate that received substantial international media coverage. The Brazilian National Bishops' Conference lobbied hard for reversal of the ruling, whereas the National Confederation of Healthcare Workers (the governmental health sector union) pushed for its permanent acceptance. In October 2004, the full Brazilian Supreme Court convened and voted 7-4 to suspend the judge's solitary ruling until the full tribunal had the opportunity to deliberate and rule on the matter. As of November 2005, the issue still had not been settled and advocates continued to debate the moral, religious, and public health implications of liberalizing Brazilian abortion laws [6-8].

Although it has been the opinions of Brazilian judges and interest groups that have made headlines in this latest chapter of the country's ongoing abortion debate, physicians that play a crucial role in the accessibility and availability of safe and legal abortion. Their opinions on abortion, their willingness to perform an abortion, and their knowledge of abortion procedures directly affect whether their patients will be able to have access to safe and legal abortion. For this reason, it is important to understand their opinions and knowledge of abortion law, their knowledge of abortion procedures and their practices related to abortion.

Several studies have documented general public opinion of abortion in Brazil. A 1989 study by Meira and Ferraz examined medical and law student opinions of abortion law and found that almost half of the students surveyed thought that the law should allow for a legal abortion in more circumstances than currently permitted by Brazilian law. The majority of medical students thought that abortion should be legal in cases of rape (96%), risk to the woman's life or health (91%), congenital anomalies (86%), and mental incapacities (63%). A smaller percentage agreed with legalizing abortion in cases where the pregnant girl was less than 14 years old (36%) [9]. A survey of 1,456 women in a Southern county in Brazil revealed that 30% were in favor of legalizing abortion regardless of the circumstances, and those with higher education, higher family income and who had previously had an induced abortion were all more likely to support legalization of abortion[10]. In contrast to these women, a qualitative study of 71 Catholic, male college students in Brazil found that they generally had a negative opinion of abortion. When asked what they would do if a woman asked their advice regarding an induced abortion, 83% said they would counsel her not to abort [11].

A 2003 study in Brazil on attitudes towards abortion compared opinions of teenage women who had aborted, women who had considered abortion but ultimately did not abort, and women who did not abort. Initially, teens who had aborted and who had considered abortion were more tolerant of abortion than those who did not abort; however, their acceptance level decreased over time. Over the one-year follow-up period, the teens that did not consider abortion became more accepting of abortion. On average, across the three groups, 66% thought that an abortion was justified when the woman's life or health was in danger, 63% thought it was justified in the case of rape, and 47% felt that it was justified in the case of congenital anomalies [12].

In terms of abortion opinions of physicians, a small study conducted among 57 emergency room physicians in two São Paulo hospitals found that 31.5% demonstrated low knowledge of Brazilian abortion laws, and the vast majority were in favor of abortion in cases of rape (84%), risk to the mother's life (86%), and fetal malformation incompatible with life (82%) [13]. More recently, in 2003, Faúndes *et al*. surveyed a national sample of 4,261 Brazilian obstetricians-gynecologists (OB-GYNs) about abortion, asking participants whether they had helped their patients or relatives to have an abortion, and whether they themselves had had an abortion. The authors found that the respondents were progressively more accepting of legal abortion the closer they were to the person with the unwanted pregnancy: 41% of respondents had helped a patient to obtain an abortion, 49% had helped a relative to obtain an abortion, 78% of female physicians had obtained an abortion when they themselves had an unwanted pregnancy, and 80% of male physicians had helped their partners to obtain abortions. The vast majority of participants believed abortion should be legal if the woman's life is at risk (79%), if the pregnancy resulted from rape (80%), and in cases of fetal malformation (77%). Younger respondents were less supportive of abortion when confronted with unwanted pregnancies of patients or relatives, and twice as many physicians with no religious beliefs had helped patients or relatives to have an abortion compared to physicians to whom religion was very important. Nevertheless, when the physician herself or the male physician's partner had had an unwanted pregnancy, almost 70% of those to whom religion was very important had had an abortion [14,15].

The relevance of Brazilian physicians' abortion knowledge, attitudes, and practices extends far beyond the current legal limbo regarding abortion of anencephalic fetuses, and we seek to build on the Faundes *et al*. findings, some of which have not been published in an English-language journal [15]. We explored not only Brazilian OB-GYNs' knowledge and opinions on the legality of abortion in their country, but also their clinical experience and training (or lack thereof) in different abortion techniques [including medical abortion and manual vacuum aspiration (MVA)] and the different circumstances (*e.g*., in cases of risk to the woman's life or when the unwanted pregnancy is the result of a rape) under which they performed abortion. Faundes *et al*. documented OB-GYN abortion practices in terms of the relationship the provider held with the pregnant women, and we will expand on these findings by investigating the circumstances under which the pregnancies arose in those cases where OB-GYNs performed abortions. Finally, we will analyze the relationships between sociodemographic characteristics and experience in performing abortions on opinions on abortion law.

## Methods

From December 2001 through September 2002, we mailed 1,500 questionnaires to a 10% random sample of OB-GYNs affiliated with the Brazilian Federation of Obstetricians and Gynecologists (FEBRASGO). At the time, FEBRASGO consisted of approximately 15,000 members. Our sample consisted of 10% of FEBRASGO members in each state, selected by assigning random digits to each member and then selecting a 10% random sample. In collaboration with FEBRASGO, we attempted to increase the response rate by using FEBRASGO stationery, publishing an advertisement in the FEBRASGO journal, sending reminder faxes, and offering a raffle. The Council's Institutional Review Board as well as FEBRASGO approved this study.

The anonymous questionnaire, which had undergone two rounds of pre-testing prior to fielding the study, contained 18 questions to assess respondents' knowledge of abortion law (specifically in cases of rape and life-threatening congenital malformations), their opinions of current abortion law in Brazil, their familiarity with various abortion procedures, and their experiences providing abortions. We also collected sociodemographic information, including sex, age, religion, and region of residence.

The primary outcome measure was opinion of abortion law. On the questionnaire, respondents were asked to choose one or more of the following circumstances in which he or she thought abortion should be legal in Brazil: life-threatening congenital malformation, rape, risk to a woman's life, risk to a woman's physical health, socioeconomic reasons, as an elective procedure, never legal, or under other circumstances. We classified respondents as conservative or liberal on abortion law based on their opinions of abortion law under the different circumstances. Conservative physicians were those who felt that abortion should only be legal under the circumstances codified in the current law (*i.e*., in rape cases or when the woman's life is in danger), or those physicians who thought the law should be more restrictive. Liberal physicians were those who felt that the current abortion law should be liberalized to allow for legal abortion under at least one other circumstance in addition to the two already permitted.

Using chi-square tests and crude logistic regression models, we determined which sociodemographic, knowledge-related or practice-related variables were associated with physician opinion. Following the bivariate analysis, we conducted a multivariate analysis of physicians' opinions of abortion law. In the initial multivariate analysis, we included variables that were associated with physician opinion of abortion law in the bivariate analysis at a p-value ≤ 0.10. We also included those variables that did not have a statistically significant relationship with opinion in the bivariate analysis, but were hypothesized to be associated with opinion based on the literature. For the final multivariate model, we used the Wald test to determine which variables were most highly associated with physician opinion of abortion law. In our final model, we include all variables that are significant at the p < 0.05 level as well as those that were marginally significant.

All analyses were conducted using SPSS statistical software version 10.0.6 and Stata, version 8.2.

## Results

Out of 1,500 questionnaires mailed, we received 572 completed questionnaires (38% response rate). As shown in Table 1, the majority of the respondents were women (56%), between 26 and 45 years old (56%), and Catholic (72%). About 52% of the respondents lived in the Southeast Region of Brazil, which includes the states of Rio de Janeiro, Minas Gerais, São Paulo, and Espirito Santo. The geographical distribution of respondents was roughly equivalent to that of the FEBRASGO membership overall [14].

**Table 1:** Sociodemographic characteristics of OB-GYNs (n = 572)

Characteristic	OB-GYNs n = 572^a^	
	n	%
Gender		
Male	252	44.1
Female	320	55.9
Age		
26–35	119	21.5
36–45	199	35.9
46–55	159	28.7
56+	77	13.9
Religion		
Catholic	414	72.4
Evangelical	43	7.6
Other religion	61	10.8
Not religious	49	8.6
Region		
South	97	17.0
Southeast	300	52.4
Northeast	87	15.2
North	17	3.0
Central	54	9.4

^a^Missing data: Age (18), religion (5), region (17).

### Knowledge

Two hundred seventy-six physicians (48%) correctly identified that abortion is legal in Brazil to save the life of a woman and in the case of rape. Also, the large majority (70%) reported awareness that the Brazilian judicial system can grant court authorization of an abortion in cases of severe fetal malformations (Table 2). Considerable confusion existed over the Ministry of Health guidelines on the gestational age limit for abortion. One quarter of physicians did not know of such a gestational age limit guideline, while 40% thought it was 12 weeks and 30% thought it was 20 weeks. In terms of abortion procedures, 86% and 90% of physicians reported knowledge of MVA and dilation and curettage (D&C), respectively. A large majority (86%) also reported knowledge of misoprostol or other prostaglandins for abortion, while few physicians knew of the mifepristone and misoprostol regimen (37%) or the methotrexate and misoprostol regimen (27%).

**Table 2:** OB-GYN knowledge related to abortion (n = 572)

Knowledge	OB-GYNs n = 572^a^	
	n	%
Correct knowledge about abortion law		
Yes	276	48.3
No	296	51.7
Familiar with MOH standards regarding rape		
Yes	404	70.6
No	160	28.0
Aware that Brazilian judiciary system may authorize abortion in cases of serious fetal anomalies		
Yes	404	70.6
No	144	25.2
Knowledge of gestational age limit for abortion		
12 weeks	227	39.7
20 weeks	174	30.4
More than 20 weeks	5	< 1.0
Never legal	12	2.1
Do not know	142	24.8
Knowledge of procedures		
Manual vacuum aspiration	493	86.2
Dilation and curettage	514	89.9
Hypertonic solutions (saline or urea)	140	24.5
Misoprostol* or other prostaglandins	493	86.2
Mifepristone + misoprostol	210	36.7
Methotrexate + misoprotol	153	26.7
Other	4	0.70

^a^Missing data: Familiar with MOH standards regarding rape (8), aware that Brazilian judiciary system may authorize abortion in cases of serious fetal anomalies (24), knowledge of gestational age limit for abortion according to law (12)

* Otherwise known as Cytotec

### Opinion

Table 3 contrasts the percentage of physicians who believed that abortion is currently legal in Brazil, according to different circumstances, with the percentage of physicians who believed abortion *should *be legal. In every circumstance except for rape, more physicians believed that abortion should be legal than believed abortion is currently legal. In the case of rape, almost all (93%) correctly identified that abortion is legal and a lesser percentage (85%) thought that it should be legal. A sizeable difference emerged in the case of severe fetal malformations, where 36% of physicians incorrectly believed that it was a legal case for abortion, while 89% thought that abortion should be legal in this case. We observed a similarly large difference when the woman's health is at risk; 7% incorrectly thought that the law currently allowed for an abortion in this case, whereas 30% of physicians thought that abortion should be legal in this case.

**Table 3:** OB-GYN knowledge and opinion of Brazilian abortion law by circumstance (n = 572)

Circumstance	Believe abortion is currently legal		Believe abortion should be legal	
	n	%	n	%
Pregnancy as a result of rape*	529	92.5	488	85.3
Risk to the woman's life*	453	79.2	493	86.2
Severe fetal malformation	205	35.8	506	88.5
Risk to the woman's health	38	6.6	180	31.5
Socioeconomic reasons	3	< 1.0	74	12.9
When the woman chooses	1	< 1.0	76	13.3
Never	12	2.1	23	4.0

* At the time of data collection, abortion was legal under this circumstance

Overall, we found that only 3% of physicians agreed with the current law that allows for abortion only in cases of rape or to save the woman's life. Most physicians (77%) thought the law should be liberalized to allow for a legal abortion under more circumstances and 17% thought the law should be more restrictive. The overwhelming majority (95%) expressed their support for public funding of abortion services (Table 4).

**Table 4:** OB-GYN opinion about abortion (n = 572)

Opinion	OB-GYNs n = 572^a^	
	n	%
Believe abortion should only be legal in cases that are currently allowed by law*		
Yes	16	2.8
No	556	97.2
Believe abortion law should be more restrictive than the current law*		
Yes	99	17.3
No	473	82.7
Believe abortion law should be more liberal than the current law*		
Yes	443	77.4
No	126	22.0
Support public funding for abortion services		
Yes	545	95.3
No	24	4.2

^a^Missing data: Believe abortion law should be more liberal than the current law (3), support public funding for abortion services (3)

* At the time of data collection, abortion was legal in cases of rape or to save the life of the mother

### Practices

In addition to sharing their knowledge and opinions regarding abortion law, physicians also reported on their abortion-related practices (Table 5). Nearly 70% had never received any training on abortion procedures. Among the 33% of respondents that had ever performed an abortion, the most common procedures were D&C (60%) and the use of misoprostol or other prostaglandins (68%) for medical abortion. While most of the respondents (73%) had performed an abortion within the first 12 gestational weeks, there was also a significant number (44%) that had performed an abortion between 13 to 20 gestational weeks. Fifty-three percent had performed an abortion in the case of severe fetal malformations, 25% had performed an abortion to save a woman's life, and 19% had performed one in a case of rape.

**Table 5:** Practices related to abortion among OB-GYNs (n = 572)

Practice	OB-GYNs n = 572^a^	
	n	%
Ever received training		
Yes	176	30.8
No	393	68.7
Ever performed an abortion		
Yes	188	32.9
No	380	66.4
Number of abortions performed in the last year*		
1–5	90	47.9
6–20	10	5.3
More than 20	5	2.7
None in the last year	83	44.1
Circumstances under which abortions were performed*		
Rape	36	19.1
Risk to the mother's life	47	25.0
Severe fetal malformation	100	53.2
Risk to the mother's health	9	4.8
Socioeconomic reasons	3	1.6
Elective	18	9.6
Other	38	20.2
Procedures used*		
Manual vacuum aspiration	18	9.6
Dilation and curettage	112	59.6
Hypertonic solutions (saline or urea)	6	3.2
Misoprostol* or other prostaglandins	129	68.6
Mifepristone + misoprostol	4	2.1
Methotrexate + misoprotol	5	2.6
Other	11	5.9
Gestational age when performed abortion*		
Before 12 weeks	137	72.9
Between 13–20 weeks	83	44.1

^a ^Missing data: Ever received training (3), ever performed an abortion (4)

* Only physicians who reported having performed an abortion answered this question (n = 188); participants could choose more than one option

We encountered several statistically significant relationships between physicians' sociodemographic characteristics and abortion-related practices with their opinion of abortion law (Table 6). In a bivariate analysis, physicians who felt the law should be more liberal were more likely to have correct knowledge of abortion law, to be familiar with the abortion law regarding severe fetal malformations, and to support public funding for abortion services (p < 0.10). Catholic physicians and Evangelical physicians were not significantly different in their abortion opinions from those who reported that they were not religious; however, physicians who reported having "other" religious affiliation had lower odds of thinking the abortion laws should be more liberal. These variables were all included in an initial multivariate analysis of physician opinion, in addition to region of residence and whether or not they had ever performed an abortion. The latter variables were included due to their hypothesized association with physician opinion and their importance in the literature. In the final model, we combined Catholic and Evangelical physicians into a single group and compared them with non-religious respondents and respondents reporting "other" as their religion. We found that Catholic and Evangelical physicians were not significantly different in their abortion opinions from physicians who reported no religious affiliation. In contrast, physicians whose religious affiliation was "other" had a decreased odds of thinking abortion law should be more liberal compared to physicians with no religious affiliation (OR 0.27 (95% CI: 0.10, 0.74). In addition, physicians who had correct knowledge regarding the status of abortion law in Brazil had a 46% higher odds of favoring a more liberal abortion law compared to those who did not have correct knowledge (OR 1.46 (95% CI: 0.96, 2.22), but this finding was not statistically significant. Finally, physicians who were in favor of public funding for legal abortion services had six times the odds of favoring a more liberal abortion law compared to those who were opposed to public funding of legal abortions (OR 6.01 (95% CI: 2.53, 14.28); however, it must be taken into account that only a small number (24) had said that they opposed public funding for legal abortions. Region of residence, correct knowledge of the law regarding abortions in cases of severe fetal malformations, and experience performing an abortion were not significantly related to physician opinion in the multivariate analysis and thus not included in the final model.

**Table 6:** OB-GYN opinions of abortion law in Brazil according to selected demographic characteristics, knowledge, and practices (n = 572)

Characteristic	Law should be more liberal		Odds Ratios and 95% Confidence Intervals	
	N 443	% 77.9	OR	95% CI
Gender				
Female	245	77.0	0.90	(0.60, 1.34)
Male	198	78.9	1 (ref)	
Age				
26–35	97	81.5	1 (ref)	
36–45	159	79.9	0.90	(0.51, 1.61)
46–55	119	75.8	0.71	(0.39, 1.28)
56+	60	79.0	0.85	(0.41, 1.74)
Religion				
Catholic	328	79.6	0.54	(0.22, 1.32)
Evangelical	31	72.1	0.36	(0.12, 1.06)
Other	38	62.3	0.23	(0.08, 0.63)
Not religious	43	87.8	1 (ref)	
Region				
South	76	78.4	1 (ref)	
Southeast	234	78.5	1.01	(0.58, 1.76)
Central	45	83.3	1.38	(0.58, 3.27)
North	12	70.6	0.66	(0.21, 2.09)
Northeast	61	70.9	0.67	(0.34, 1.32)
Knowledge of abortion law				
Yes	217	74.1	1.58	(1.06, 2.37)
No	226	81.9	1 (ref)	
Familiar with MOH standards on sexual violence and abortion				
Yes	317	78.7	1.15	(0.74, 1.77)
No	122	76.3	1 (ref)	
Knowledge of gestational age limits on abortion in cases of rape				
Yes	264	79.8	0.76	(0.51, 1.13)
No	170	74.9	1 (ref)	
Surgical methods known				
Yes	431	78.6	2.05	(0.67, 6.22)
No	9	64.3	1 (ref)	
Medical methods known				
Yes	402	78.8	1.37	(0.72, 2.62)
No	38	73.1	1 (ref)	
Knowledge of law regarding fetal malformation				
Yes	325	80.6	1.67	(1.08, 2.60)
No	102	71.3	1 (ref)	
Received training				
Yes	137	77.8	0.99	(0.65, 1.52)
No	305	78.0	1 (ref)	
Support public funding for abortion services				
Yes	434	79.9	6.64	(2.83, 15.57)
No	9	37.5	1 (ref)	
Ever performed an abortion				
Yes	150	80.2	1.21	(0.78, 1.86)
No	292	77.0	1 (ref)	

## Discussion

### Knowledge

Although abortion is legal in Brazil to save a woman's life and in cases of rape, Brazilian women have limited access to safe abortion services. Two recent articles ascribed a portion of this limited access to legal abortion in Brazil to a lack of knowledge of abortion law in the general population [16,17]. Indeed, in our survey of Brazilian OB-GYNs, about half of the physician respondents could not correctly identify the Brazilian abortion law. Additionally, a significant number of physicians thought that abortion was legal in the case of severe fetal malformations when in fact, at the time of data collection, women were required to solicit permission from the judicial system for an abortion in these cases. The large number of physicians who were not familiar with Brazilian abortion law is alarming because these OB-GYNs will be unable to give accurate information to their patients. The participants' confusion may now be exacerbated by the recent turmoil surrounding abortion of anencephalic fetuses. Our findings are similar to those of Faundes *et al*., who observed that two-thirds of Brazilian OB-GYNs wrongly believed that a judicial order is required to obtain a legal abortion and only 27% knew that the woman needed to make a written request to obtain a legal abortion [15].

The majority of OB-GYNs surveyed reported familiarity with manual vacuum aspiration (MVA) and the dilation and curettage (D&C) procedures. Additionally, a large majority of physicians knew of the possibility of using misoprostol or other prostaglandins to induce abortion. Misoprostol, which is marketed under the name Cytotec, has received significant press coverage in Brazil as the drug's worldwide notoriety as an abortifacient first began in Brazil in 1988 [18]. However, fewer OB-GYNs surveyed knew of the use of misoprostol in combination with either mifepristone or methotrexate, which are more successful regimens for induced abortion [19-21].

### Opinion

In terms of opinions on abortion law, very few physicians agreed with the current law, mostly due to the fact that an overwhelming majority thought that abortion should be legal in the case of severe fetal malformations (*i.e*., they felt abortion should be legal without needing judicial authorization). As an illustration of this support, out of the 188 physicians that reported having performed an abortion, over half of them said they had performed an abortion because of severe fetal malformations. In addition, there was some support for abortion being legal when the woman's health is at risk, for socioeconomic reasons, and when a woman chooses.

Overall, OB-GYNs who had correct knowledge of abortion law, who reported to be Catholic, Evangelical, or not religious (as compared to "other" religion), and who supported public funding of legal abortion services were more likely to be in favor of a more liberal abortion law. A limitation of our study in terms of this particular finding is that respondents did not specify what their religion was if it fell under the "other" category, so we cannot hypothesize about why these participants would have more conservative attitudes about abortion. Finally, contrary to *a priori *hypotheses, physicians who had experience in conducting abortions were not more likely to support abortion being legal under additional circumstances than those who had not performed an abortion.

### Practices

About a third of OB-GYN respondents reported ever having performed an abortion. Thirty-six (19%) of those respondents who had ever performed an abortion reported doing so in the case of a rape, and 47 (25%) had performed an abortion in the case of risk to the woman's life, both of which were circumstances under which abortions were legally permitted. Nevertheless, while not specifically allowed by the law at the time of data collection, 100 (53%) of the physicians who reported ever having performed an abortion said that the abortion was a case of severe fetal malformation. It is striking that, among those respondents who had ever performed an abortion, most had done so under circumstances not explicitly permitted by law at the time of data collection.

D&C and misoprostol alone (or other prostaglandins) were the two most commonly used procedures for inducing abortion among the OB-GYNs surveyed. Very few physicians reported using MVA, despite wide recognition of its effectiveness and its increased safety and cost effectiveness over D&C [22,23]. Similarly, few physicians reported using misoprostol in combination with either mifepristone or methotrexate, even though these are more effective regimens than using misoprostol alone [19-21].

## Conclusion

The majority of OB-GYNs in our study and in the previous Faundes *et al*. study agreed that abortion should be legal when the pregnancy endangers the life of the woman or is a result of a rape (in those circumstances abortion is permitted under current Brazilian law)[14,15]. However, among those OB-GYNs with previous experience performing abortions, more than half of them had done so in cases of fetal malformation, a circumstance that was not explicitly permitted under current abortion law. Not only do these findings underscore the widespread confusion and misperceptions surrounding abortion law, but they also provide powerful evidence of the fact that abortions are being performed by OB-GYNs even in cases that are technically illegal. This information is particularly valuable to those advocating a liberalization of abortion law to permit legal abortions in cases of fetal malformation. This study also demonstrates the need for training in surgical and medical abortion techniques for Brazilian OB-GYNs. MVA is not as well known as D&C, even though the former is both more effective and less expensive than the latter. Likewise, training on medical abortion is needed to educate physicians on the benefits of using misoprostol in conjunction with mifepristone or methotrexate. Educational efforts, ideally beginning with medical trainees in universities, should focus on introducing these safer, less costly abortion techniques in Brazil. In conclusion, we hope this study can inform educational campaigns for Brazilian OB-GYNs in order to clarify misperceptions of abortion law and increase their technical capacity to provide safe, legal abortions.

## Competing interests

The author(s) declare that they have no competing interests.

## Authors' contributions

JD and SGG conceived of the study and participated in its design and coordination. LAG, SGG, and EAY performed data analysis and helped draft the manuscript. All authors read and approved the final manuscript.

**Table 7:** Adjusted odds ratios of the likelihood of thinking that the abortion law should be more liberal (n = 572)

Characteristic	Odds Ratio	95%CI	p-value
Religion			
Catholic/Evangelical	0.63	(0.27, 1.52)	0.301
Other religion	0.27	(0.10, 0.74)	0.011
Not religious	1 (ref)		
Correct knowledge of abortion law			
Yes	1.46	(0.96, 2.22)	0.077
No	1 (ref)		
Support public funding			
Yes	6.01	(2.53, 14.28)	< .001
No	1 (ref)		
